# Microplastic pollution in Himalayan lakes: assessment, risks, and sustainable remediation strategies

**DOI:** 10.3762/bjnano.16.148

**Published:** 2025-11-25

**Authors:** Sameeksha Rawat, S M Tauseef, Madhuben Sharma

**Affiliations:** 1 Sustainability Cluster, School of Advanced Engineering, UPES, Dehradun, Uttarakhand, India; 2 Research & Development, UPES, Dehradun, Uttarakhand, India

**Keywords:** biofilms, freshwater system, Himalayan lakes, microplastic pollution, nanotechnology, remediation

## Abstract

Microplastic contamination is a newly emerging environmental problem in the ecologically sensitive Himalayan lakes, posing a threat to biodiversity, water quality, and human habitation. These high-altitude freshwater ecosystems are being increasingly polluted through human use, tourism, glacier melt, and atmospheric deposition. Microplastic quantification in such isolated locations is, however, limited by factors such as harsh climatic conditions, logistical challenges, and the need for expert analytical techniques like microscopy and spectroscopy. The present review considers sources, pathways, and ecological impacts of microplastics in Himalayan lakes compared to other sensitive aquatic ecosystems. The review describes existing remediation technologies, categorizing these into physical, chemical, and biological interventions, and takes into account emerging sustainable approaches, including biofilm-mediated degradation and nanotechnology-based solutions. The application of nanomaterials for microplastic removal is elaborated in detail, and case studies validated their effectiveness, especially in cold environments with strong UV irradiation. In the face of increasing worldwide research into microplastic contamination, there remains a huge knowledge gap concerning its behavior in distant, elevated lake systems such as the Himalayas. The most important areas to focus with regard to the ecotoxicological impact of microplastics are the bioaccumulation of microplastics in the Himalayan food web, plasticizer toxicity, and long-term potential health and ecological threats. This review addresses the imperatives of enhanced governance, monitoring, legislation, and community-based mitigation measures. This research makes a contribution by integrating region-specific data, defining priority research needs, and provoking sustainable, multidisciplinary solutions specific to freshwater cold-climate ecosystems. This contribution serves to address the imperative of adopting multidisciplinary research, region-specific remedial measures, and judicious estimation of microplastic contamination of high-altitude lakes through by describing research gaps. It distills the present scenario and promotes novel, environmentally friendly remedial measures, regulatory policies, cooperative initiatives to combat microplastic pollution, and vulnerabilities in the fragile Himalayan freshwater aquatic ecosystems.

## Review

### 1 Introduction

The Himalayan lakes, often known as the “Water Towers of Asia,” serve an important role in biodiversity conservation, downstream hydraulic management, and supplying freshwater supplies to millions of people. Sau et al. explain that Pangong, Tsomoriri, and Tsokar are typical instances of untouched alpine lakes that support indigenous species and lifestyles [1]. The Nainital lakes, including Naini Lake and Bhimtal Lake, are crucial to the region’s environment and tourism. The unique ecosystem of the lakes and rising anthropogenic pressures have made them a target for environmental research [2]. Similarly, the Ramsar wetland Loktak Lake is renowned for floating vegetation and being a sanctuary for the endangered Sangai deer [3]. These alpine lakes are exposed to increasing environmental stresses due to human population, population increase, and climate change [4]. Himalayan lakes vary widely and include, for example, the Pangong Lake (Ladakh, high-altitude, saline), the Nainital Lake (Uttarakhand, mid-altitude, urban-influenced), and the Dal Lake (Kashmir, low-altitude, densely populated). Their altitudes, catchment characteristics, and anthropogenic pressures result in distinct pathways of contamination with microplastics (MPs).

MP contamination is currently a problem of global proportions impacting freshwater, oceanic, and terrestrial ecosystems. Although initial studies primarily addressed oceanic ecosystems, current studies show the increasing load of MPs in inland water bodies, for example, rivers, lakes, and estuaries [5]. For South Asia, especially, high MP loads in major river systems such as the Ganges and Brahmaputra have all been driven by rapid urbanization, poor plastic waste management, and hydrological connectivity [6]. Yet, although the upland catchments are so crucial to maintaining these rivers, high-altitude regions, above all, the Himalayas, are still inadequately studied. Himalayan lakes are exposed to increased plastic contamination by natural and human-induced processes. The complex process of MP transport and deposition is outlined in Section 3. The harsh environmental conditions of the region, freeze–thaw weather, low microbial activity, and short hydrological retention times, make the degradation of MPs more difficult and these lakes to long-term sinks for plastic particles.

Himalayan lakes are very susceptible to MP pollution [4,7–9]. MPs enter these ecosystems due to glacier melting, tourism, agricultural runoff, and inadequate waste management. Anchar Lake and Dal Lake in Kashmir, for instance, exhibit high MP levels owing to household waste and touristic activities [10]. Recreational activities and urban runoff have resulted in plastic pollution increasing in Nainital Lake, a popular tourist destination, threatening aquatic biodiversity [2]. Nazir et al. state that MPs pollute water bodies by accumulating in food chains and bearing toxic contaminants such as heavy metals. This problem is exacerbated by the fact that plastic pollution has a transboundary implication, thereby having the chance of ending up in other lakes’ sediments and water [11].

The existence of MPs in both Indus and Brahmaputra rivers suggests that the pollution from the highland lake and glacier may reach into downstream water systems [12]. According to Kumar et al., the distribution and accumulation of MP are considerably influenced by anthropogenic activities within catchment areas, which is further worsened by inadequate waste management systems. This is evident in studies conducted on lakes such as Nainital and Manasbal [2,9,13]. This review compiles recent evidence on MP contamination of Himalayan lakes and contrasts it with global high-altitude ecosystems. It will propose to identify special regional vulnerabilities, evaluate existing limitations in monitoring and remediation, and suggest strategies specific to vulnerable, cold-climate aquatic ecosystems. It tries to identify research gaps, providing practical frameworks for policymakers and researchers to protect such vulnerable ecosystems and regulate MP pollution.

### 2 Methodology

This is a systematic review article with the objective of consolidating the existing literature on freshwater MP pollution in Himalayan high-altitude lakes and groundwater ecosystem. Relevant literature was retrieved from ScienceDirect and Google Scholar, using the combinations of the following keywords: “microplastics”, “freshwater lakes”, “Himalayas”, “glacial lakes”, “groundwater contamination”, “plastic toxicity”, “MP remediation technologies”, “bioremediation”, “SDG 6”, and “ecological risk”.

**Figure 1:**
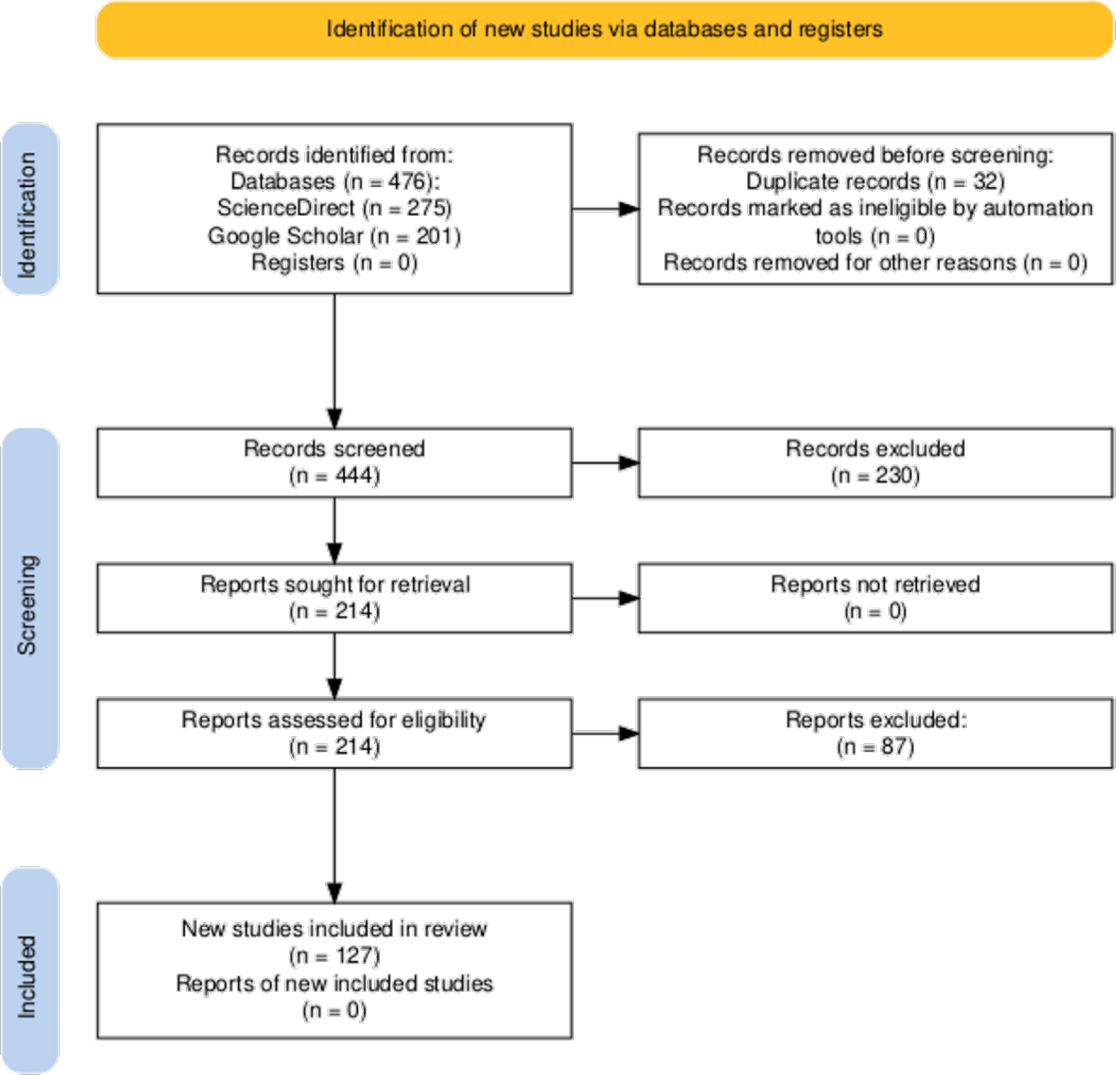
PRISMA flowchart of literature search and selection process [14].

Literature search was restricted to articles published in the period of 2010–2025. Peer-reviewed journal articles, book chapters, and technical reports published in the English language were included. Excluded were marine-only studies, conference abstracts, editorials, and non-peer-reviewed articles. A total of 476 records were identified. Removing duplicates and irrelevant items after title and abstract screening left 127 studies for the final synthesis. In order to facilitate transparency, a “Preferred Reporting Items for Systematic Reviews and Meta-Analyses” (PRISMA) flow diagram (Figure 1) is provided to describe the article selection process.

### 3 Vulnerability of Himalayan lake ecosystems to microplastic pollution

This section presents the environmental setting and vulnerability context of Himalayan freshwater ecosystems to set the stage for the following assessment of MP pathways, effects, and mitigation measures. The Himalayan region has more than 16,000 alpine and glacial lakes, most notably being crucial reservoirs of freshwater for tens of millions of people downstream [15]. Himalayan alpine lakes are environmentally sensitive because of their glacial origin, steep altitude gradients, and high hydrological sensitivity to rapid climate change [16–17]. It is important to know the distinct environmental characteristics and sensitivities of Himalayan alpine lakes to place the mechanisms and deposition of MPs into sensitive environments in perspective [18–19].

#### 3.1 Sources and pathways of microplastics in lakes

MPs come from various sources and are carried to high-altitude lakes in the Himalayas through several routes. The major sources of pollution are improper disposal of garbage, uncontrolled tourism, and agricultural runoff. For instance, Kashmir lakes, including Dal and Anchar, are extremely polluted due to their proximity to human dwellings and the litter that is created due to tourism [4]. Recent studies confirm the occurrence of MPs in Himalayan high-altitude lakes due to surface runoff from tourist activities, plastic trash, and unregulated effluent. For instance, Jain et al. reported a MP density of 110–370 particles/m^3^ in the Nainital Lake, Uttarakhand, which was highest during the tourist season. The outcomes confirm that human accessibility and lake morphology (glacier-fed and rain-fed) influence MP density, though this interaction is poorly studied and requires further work [2]. Similar to what has been happening in Ladakh’s Pangong Lake, MPs carried in the air and over long distances are deposited in water bodies via atmospheric deposition. This is a key mechanism in remote lakes besides direct input [7].

Groundwater modeling methods have been instrumental in describing the transport and fate of contaminants, including MPs, in subsurface environments. Numerical methods such as MODFLOW coupled with transport methods such as MT3DMS and RT3D are widely used to simulate groundwater flow and contaminant transport processes [20]. Such models have been adapted to cold mountain environments by adding glacier-fed recharge zones, freeze–thaw processes in soils, and topography-induced flow [21]. Semi-distributed models such as SWAT and MIKE SHE have also been used regarding Himalayan catchments to simulate subsurface flow and contaminant loading under varying climate and land use scenarios [22]. The models can be used to simulate MP transport in high-altitude lake basins, although particle-bound adaptations are under development.

**Figure 2:**
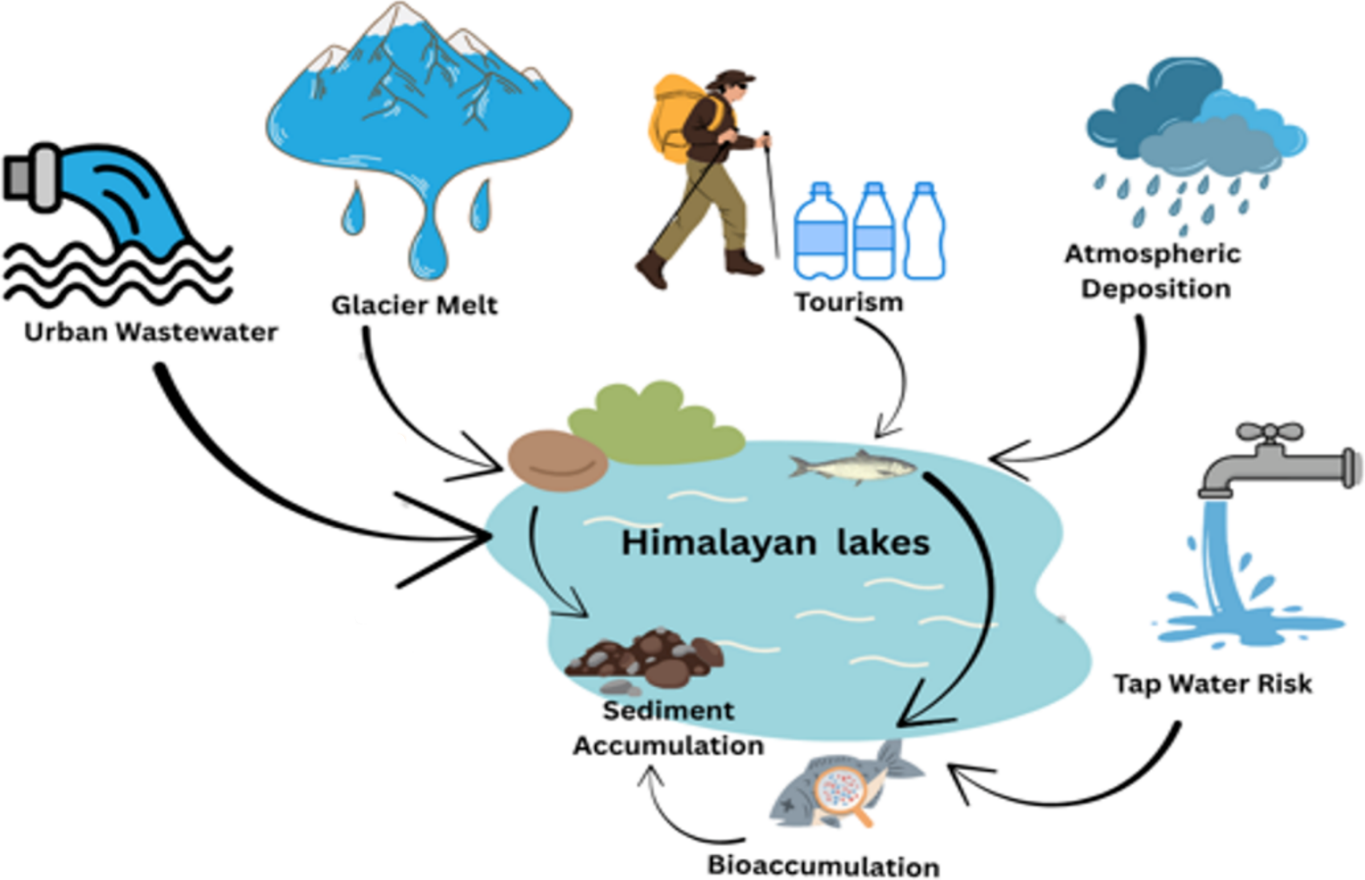
Infographic showing the lifecycle of MPs in Himalayan lakes. Graphical element Wastewater Vector Icon Design: © 123 Stock via Canva.com; Graphical element Melting Glacier Illustration: © Goldink Studio via Canva.com; Graphical element Hiker with Walking Sticks and Backpack: © Vanessa E. E. via Canva.com; Graphical element: Plastic Waste Blue: © Karyative via Canva.com; Graphical element Raining Cloud: © piyanuch28 via Canva.com; Graphical element Running Tap Water, Simple Illustration of Water Flowing from a Faucet: © mike223via Canva.com; Graphical element Lake element: © Visula Co via Canva.com; Graphical element Fish: © Canva Creative Studio via Canva.com; Graphical element Microplastics in Fish Illustration:© Azharialr via Canva.com; Graphical element Pile of Sediments: © Canva Creative Studio via Canva.com; Graphical element Curved thin doodle hand drawn arrow left: ©Visual Generation via Canva.com; Graphical element Curved thin doodle hand drawn arrow right: © Visual Generation via Canva.com.These elements are not subject to CC BY 4.0.

The melting of glaciers has been identified as a distinct mechanism for MPs in the ecosystems of the Himalayas. MPs trapped in glacier ice are released as it melts, subsequently entering freshwater streams and lakes downstream [23]. Recently sampling in Pangong Lake and Beas catchment [2] has linked MP presence to glacial retreat zones, affirming meltwater pathways. Where Himalayan-specific data is sparse, mechanisms are inferred from analogous systems such as the Swiss Alps and Andean lakes. Seasonal MP dynamics in the Himalayan lakes are controlled by environmental factors such as glacier melting and monsoonal runoff. Summer melt pulses enhance the transport of MPs from catchment basins, whereas weak winter flows allow for sedimentation. Perennial flow of the Brahmaputra and the Koshi rivers across borders makes sure that extraneous contaminants enter such pristine ecosystems [24]. Hydrological conveyance is also seen in research in the Beas catchment, where MPs in low areas are transported upwards into sensitive ecosystems [25]. Such connectivity of the routes and sources is apparent in Figure 2, which presents the wide ecological extent and conveyance of MPs in water bodies. To allow aggregation of regional results, Table 1 reports documented MP density in some Himalayan freshwater lakes and their typology. Table 2 is a compilation of the most commonly reported dominant polymers and morphologies, as well as the size classes, of MPs in Himalayan lakes.

**Table 1:** Documented MP concentrations in Himalayan lakes.

Lake	Location	Lake type	Segment	MP concentration		Avg. MP concentration		Ref.
				Surface water [SW] (particles/m^3^)	Sediment [S] (items/kg dw)	Surface water (particles/m^3^)	Sediment (items/kg dw)	

Dal	Jammu and Kashmir	spring-fed	SW + S	–	–	196650 ± 350	416 ± 38	[11,26]
Manasbal	Jammu and Kashmir	spring-fed	SW + S	13000–89000	840–4020	–	–	[9]
Nainital	Uttarakhand	rain-fed	SW + S	8600–56000	400–10600	–	–	[2]
Rewalsar	Himachal Pradesh	spring-fed	SW + S	13000–238000	750–3020	–	–	[27]
Anchar	Jammu and Kashmir	river-fed	S	–	233–1533	–	660 ± 360	[10]
Renuka	Himachal Pradesh	spring-fed	SW + S	2000–64000	15–632	–	–	[28]

**Table 2:** Common MP characteristics including dominant polymers and morphologies, and size classes in Himalayan lakes.

Lake	Dominant polymers	Dominant morphologies	Size class (µm/mm)	Reference

Dal	PE, PS, PP, PVC	fragments, fibers, pellets	<250 µm	[26]
Manasbal	PP, PE, PS	beads	–	[9]
Nainital	HDPE	fibers	0.02–1 mm	[2]
Rewalsar	PS, PE, PP	pellets, fragments	–	[27]
Anchar	PA (96%), PET (1.4%), PS (1.4%), PVC (0.9%), PP (0.7%)	fibers (91%), fragments/films (8%), pellets (1%)	–	[10]
Renuka	PE, PS	fibers, fragments	–	[28]

#### 3.2 Impact on biodiversity, water quality, and human communities

The presence of MPs in water systems significantly affects the quality of water, biodiversity, and human well-being. Studies have confirmed the ingestion of MPs by aquatic organisms, including fish, mollusks, and crustaceans in Lake Nundkol and Dal. This renders aquatic animals highly vulnerable to the action of plasticizers. Consumption of these substances interferes with metabolic processes and leads to bioaccumulation of toxic compounds, like heavy metals linked with MPs [11]. Altitude lake sediments contain high concentrations of MPs, which interfere with benthic assemblages and influence the food web structure [4]. For instance, a study in Dal Lake recorded MP concentrations of 2450 particles/m^2^ in sediments and 9.8 particles/L in surface water [11], indicating significant ecological exposure.

#### 3.3 Global comparison with other sensitive aquatic ecosystems

Across the globe, MPs have emerged as a widespread contaminant in delicate aquatic ecosystems, and Himalayan lakes exhibit numerous similarities with those found in other high-altitude or pristine environments. For example, MPs from glacial melt and atmospheric deposition cause problems for lakes in Europe’s Alpine regions, especially those in the Swiss Alps [23]. However, MPs degrade more slowly in the Himalaya’s cooler climates, which over time makes their buildup more damaging and permanent.

In South America, the Andean lakes exhibit comparable MP pathways, such as agricultural runoff and tourism, yet they vary in management approaches. According to Yang et al., systematic management methods are often not applied to the Himalayan lakes; therefore, there is a greater risk of ecological breakdown [24]. In contrast, the Andean lakes benefit from community-based rehabilitation processes. The Beas River in the Western Himalaya has been compared with rivers in temperate zones, where improved waste management systems cause a reduction in MP accumulation. According to Bhaduri et al., this disparity reflects how essential cooperating waste management policies and plans are in the Himalayas to prevent MP pollution [25].

Matta et al. provided a comprehensive historical analysis of water and emerging pollutants, specifically microplastics, in the Indian Himalayas, highlighting how such pollutants have progressively affected surface and groundwater as a result of unregulated use of plastic and a lack of waste management infrastructure [29]. Our research supplements theirs by emphasizing not just the presence and ecological hazards but also possible remediation technologies and SDG-consistent governance options. In addition, Nayak et al. employed pollution indices and chemometric models to assess the presence of poisonous elements in Himalayan groundwater [30]. Our addition of groundwater vulnerability modeling and toxicity pathways to this contributes by incorporating MPs into groundwater threat frameworks, providing a new contribution in linking solid plastic pollution with hydrological threats in vulnerable highland environments.

### 4 Methods for assessment of microplastics

#### 4.1 Sampling strategies in remote regions

MP sampling in remote mountainous areas at high altitudes, for example, the Himalayan lakes, necessitates creative logistics and solutions adapted to environmental conditions. The conditions in these areas generally render conventional methods, for example, trawling plankton nets in surface water, infeasible owing to transport and size constraints of equipment [31]. This has given rise to sampling devices that are both portable and light, specifically tailored for use in remote environments. For example, surface MPs can be effectively collected in streams and lakes by adapting small mesh nets (100–300 µm).

The collection of sediment samples is also essential since MPs have a tendency to sink and build up over time. The most effective methods for recovering sediments from lakebeds in waters of shallow to medium depth are grab samplers and hand-held corers [32]. MP deposition greatly depends on glacial runoff and alpine seasonal tourist activities, such that seasonal sampling protocols need to be adopted. While the monsoon season in the Himalayas typically spans from June to September, this review emphasizes late-monsoon periods (August–September) due to the heightened runoff and peak tourism activity during these months. Sample collection both before and after the monsoons could potentially provide useful insight into variations in contamination levels generated due to runoff and anthropogenic activities.

**Figure 3:**
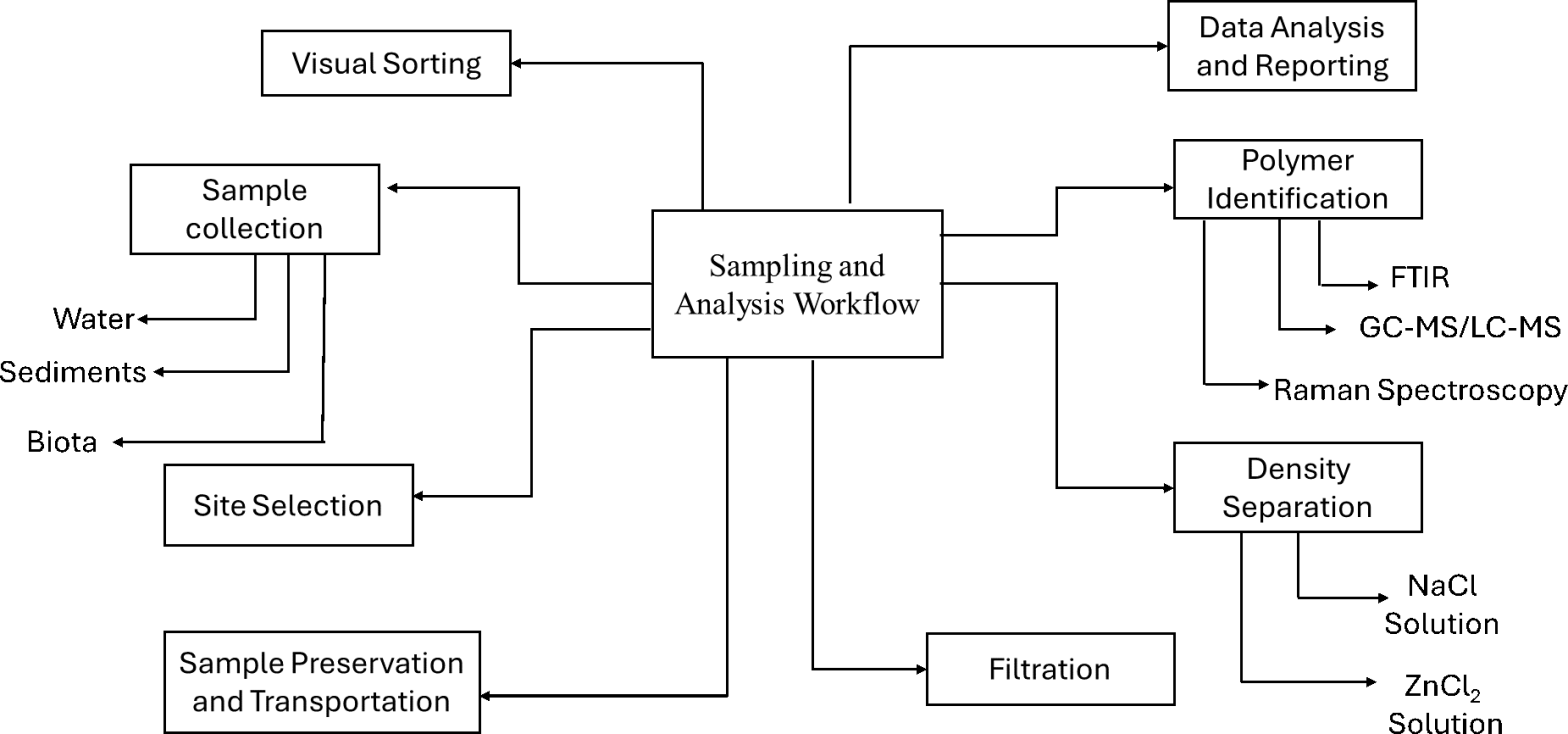
Sampling and analysis methods for MPs in remote regions.

The collection of both suspended and floating particulates is facilitated by the increasing use of in situ filtration systems that utilize portable vacuum compressors for water samples. These tactics aid in capturing MPs of all sizes, from the macro- to the nanoscale, which is essential for understanding the full contamination profile in remote ecosystems [33]. An overview of MP sampling and analysis methods is given in Figure 3.

#### 4.2 Analytical techniques for microplastic characterization

**4.2.1 Spectroscopy.** One of the key methods of analyzing MPs is spectroscopy. Fourier-transform infrared (FTIR) spectroscopy is used regularly to identify the chemical structure of MPs [34]. One of the most important developments for nanoplastic detection in complex environmental matrices is micro-FTIR to analyze particles smaller than 10 µm [35].

Raman spectroscopy improves on FTIR by using higher resolution and the ability to examine colored or pigmented polymers without dye interference. The geographical distributions of MPs in samples are increasingly being mapped using Raman mapping techniques, which provide valuable information for ecological impact studies [36]. Such techniques are able to distinguish between polymers that result from local tourist waste and those transported by atmospheric deposition in Himalayan lakes of high elevation [37].

**4.2.2 Microscopy.** Microscopic techniques remain crucial for the initial description of MPs, particularly when assessing their physical properties. Scanning electron microscopy (SEM) produces high-resolution images of particle shapes. It also indicates surface wear and tear patterns that reflect how old the MP is and how long it has been in the environment [38].

Ease of operation and minimal cost of optical microscopy make it widely used, despite being less sophisticated than SEM. It is particularly useful in morphological examination, size grading, and rapid particle identification. The combination of optical microscopes and modern digital imaging techniques allows for automatic particle classification and counting, which greatly decreases the extent of human intervention and possibility of error [39].

**Figure 4:**
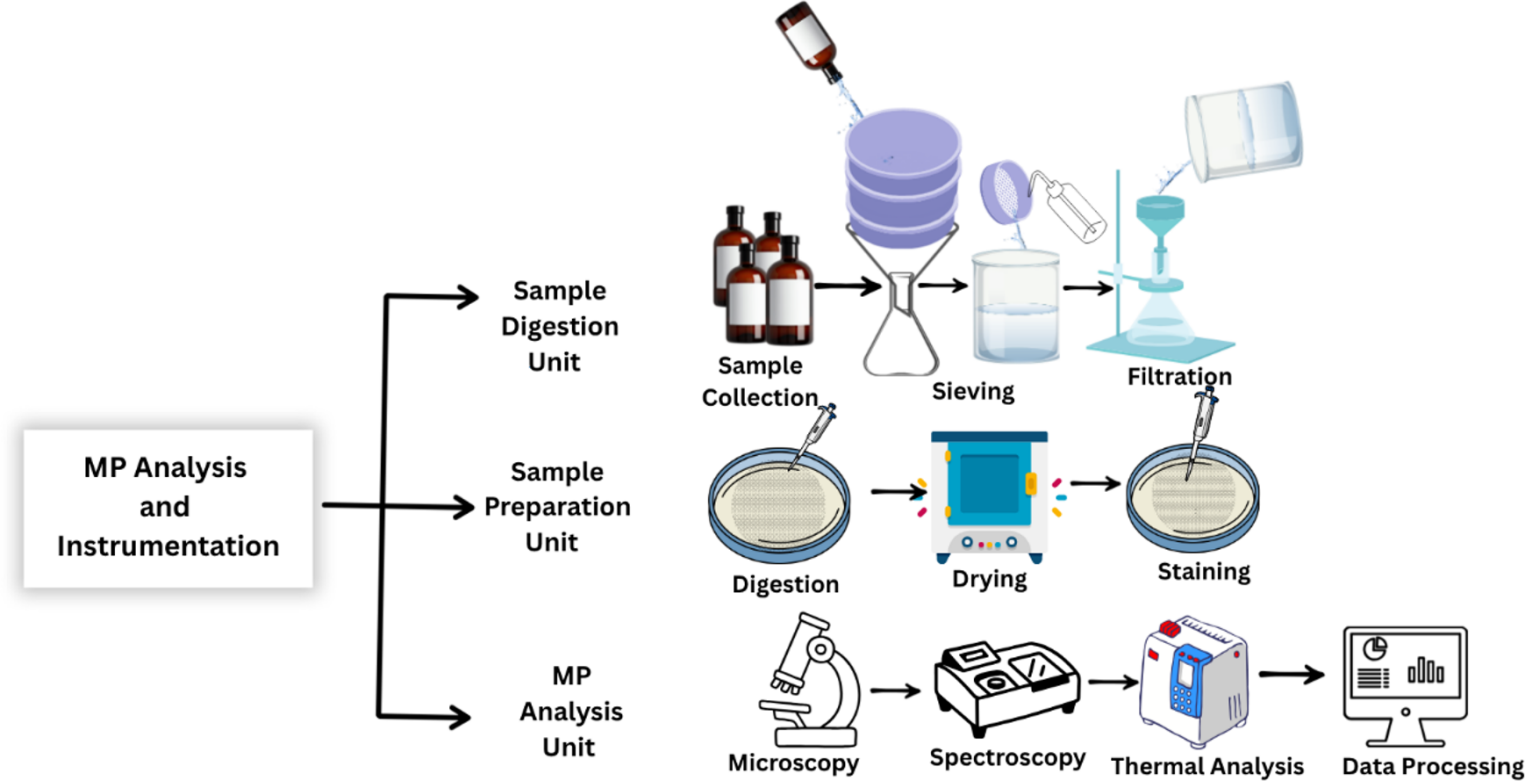
Diagram of MP analysis instrumentation. Graphical element Amber glass bottle with blank label: © andiiwan via Canva.com; Graphical element Laboratory Conical, Titration or Erlenmeyer Flask: © Vector tradition via Canva.com; Graphical element Filter funnel icon: © musmellow via Canva.com; Graphical element Flour Sieve Illustration: © ivandesign via Canva.com; Graphical element Splash of Water: © Billion Photos via Canva.com; Graphical element Transparent beaker with clear water on white background: © BlueRingMedia via Canva.com; Graphical element Wash Bottle: © awspicious via Canva.com; Graphical element Clean Flat Vacuum Filtration Apparatus: © sparklestroke via Canva.com; Graphical element Petri Dish with Cream-Colored Agar Illustration: © Maria Teruya's Images via Canva.Com; Graphical element Round Grid Filter Outline Thin Line Style © Serhii Borodin via Canva.com; Graphical element pipette: © heyrabbiticons via Canva.Com; Graphical element incubator: © putra-creative via Canva.com; Graphical element Microscope Outline School Supplies: © Langgeng Pangrebowo via Canva.com; Graphical element Spectrophotometer: Vectors Market via Canva.com; Graphical element Gas Chromatograph: © Aurielaki via Canva.com; Graphical element Monitor: © Ibrandify via Canva.com; Graphical element White Rectangle With Shadow: © StylishDesignStudio via Canva .com; Graphical element Triple split arrow in linear style: © Nazar12 via Canva.com; Graphical element Arrow Down Icon: © Noun Project (limited collection) via Canva.com. These elements are not subject to CC BY 4.0.

Microscopy and spectroscopy tend to be combined to increase reliability. For instance, Raman or FTIR spectroscopy is applied to identify polymers following SEM to examine the shape of particles. This ensures both chemical and physical characteristics are captured [40]. The combined analytical flow and instrumentation for MP characterization, such as digestion, preparation, and analytical processes like microscopy, spectroscopy, and thermal analysis, is presented in Figure 4.

#### 4.3 Challenges in quantification and identification under alpine conditions

MPs assessment is particularly problematic at high altitudes, including the Himalayas. Implementation of sophisticated analysis methods and sampling is limited due to extreme climatic conditions like below-freezing temperatures, strong UV irradiation, and poor infrastructure [31]. Portable and robust field-friendly technologies are also required since it is challenging to transport samples to well-equipped laboratories from distant locations.

Another challenge is that natural particulate matter such as minerals, biological debris, and glacial silt can lead to the interference of MP detection. MPs must be separated by using pretreatment techniques such as enzymatic decomposition of organic matter and density separation using saline solutions. These processes, however, might take a lot of time and could cause tiny particles to be lost [32].

The distinct combination of nanoscale dimensions and very low concentration in samples from the environment makes nanoscale plastics yet another challenge. Sophisticated methods such as thermal extraction desorption (TED) and gas chromatography-mass spectrometry (GC-MS) are now routinely used to detect these particles. But these methods involve a lot of expertise and equipment. The development of portable, low-cost systems for nanoscale analysis is a significant field of future studies [34].

### 5 Remediation techniques

#### 5.1 Overview of current technologies for microplastic removal

Various physical, chemical, and biological methods are employed with the hope of effectively removing MPs from water bodies. The simplest process to remove MPs from water supplies is by physical processes. Filtration processes, including sand filtration and membrane bioreactors (MBRs), utilize size exclusion to remove MPs [41]. Tiwari et al. established that specifically MBRs have been highly efficient, with more than 99% removal efficiencies under controlled conditions [42]. Sedimentation methods are frequently used in wastewater treatment plants, making use of the density difference between MPs and water. But it is difficult to trap particles measuring less than 10 µm with such processes. Physical methods are easy to implement and scalable but lack efficiency in open, natural environments such as Himalayan lakes and often require energy-consuming setups [43]. Research also indicates that sophisticated filtering systems, including granular activated carbon (GAC) filters, effectively capture small MPs. They are therefore ideal for use in high-altitude lakes [44].

The goal of chemical methods for MP remediation is to convert polymers into non-toxic substances. Advanced oxidation processes (AOPs) use strong oxidants like ozone, hydrogen peroxide, or hydroxyl radicals to break down MPs [45]. The most promising among them are photocatalytic processes that utilize titanium dioxide nanoparticles under UV radiation. These are very efficient degradation processes for degrading plastics to carbon dioxide (CO_2_) and water [43]. Fenton reactions, involving a mixture of hydrogen peroxide and iron catalysts, are a good example of a new method that has achieved great degrading effectiveness and is under study regarding its potential application to cold environments [46]. However, before being widely used in delicate ecosystems like Himalayan lakes, chemical methods must be carefully optimized since they frequently demand substantial energy input and can produce secondary pollutants [47].

Biological processes can utilize the natural ability of microorganisms to degrade synthetic polymers. Microorganisms such as *Pseudomonas* and *Bacillus*, which have enzymatic activity, can degrade plastics into smaller molecules [48]. Biofilms adhering to the plastic surfaces are required for biological degradation since they secrete enzymes that degrade polymers outside cells [49]. As noted by Rai et al., biofilms can speed up the breakdown of MPs, even though such a process is prone to interference from environmental factors like pH, temperature, and the availability of nutrients [50]. There has been promising development within the field of genetic engineering in relation to the creation of microbial consortiums optimized for high-efficiency degradation [46]. As reported by Rizvi et al., some fungi, such as *Aspergillus spp.*, have already shown potential in degrading MPs in a controlled settings, with the possibility of further application in larger-scale bioremediation schemes [44].

Table 3 gives a comparative summary of the most recent physical, chemical, and biological technologies for MP remediation. Among them are new electrochemical technologies, membrane separation technologies, microbial technologies, and AOPs. The efficiency, mechanism, strengths, weaknesses, and applications of each technology are utilized to assess each technology.

**Table 3:** Current technologies for MP removal.

Technology	Mechanism/type^a^		Advantages	Limitations	Efficiency	Applications	Ref.

piezocatalytic and Fenton-like H_2_O_2_ activation using Bi_12_(Bi_0.5_Fe_0.5_)O_19.5_	synergistic oxidation and fragmentation of polyethylene terephthalate (PET) MPs via reactive oxygen species (ROS) generation	C	effective degradation of PET-MPs (28.9% removal in 72 h); enhanced efficiency compared to individual Fenton or piezocatalysis; durable catalyst with multiple reusability	slow degradation rate compared to adsorption or filtration methods; requires ultrasound treatment and optimized oxidant concentrations	28.9% removal for PET-MPs (10 g/L) in 72 h	industrial wastewater treatment and environmental remediation	[51]
capillary skimming using hydrophilic ratchet	capillary force-driven skimming at the air–water interface	P	effective for floating MPs (1–4 mm) with densities from 0.02 to 0.97 g/cm^3^; avoids clogging issues in mesh-based filters; demonstrated feasibility with a marine robot cleaner	limited to floating MPs, ineffective for submerged or sinking MPs; dependent on water bridge stability for efficiency	high efficiency for floating MPs across different densities	marine pollution control and robotic surface-cleaning systems	[52]
settling treatment in wastewater treatment plants (WWTPs)	sedimentation based on density, size, and shape of MPs	P	effective for MPs with higher density and larger size; predictable efficiency based on settling models; commonly integrated into existing wastewater treatment processes	lower efficiency for small, low-density MPs; removal efficiency depends on surface loading rate and operational conditions	variable efficiency based on MP density, size, and shape	wastewater treatment plants and industrial effluent management	[53]
electrochemical oxidation with boron-doped diamond electrodes	indirect oxidation of MPs via reactive radicals	C	effective degradation of 1.0 µm polystyrene (PS) MPs; achieves oxidation and morphological changes in MPs; uses highly oxidizing radicals for degradation	requires optimization of power usage and electrode cost; long treatment time (5 h)	high efficiency under optimal conditions (Na_2_SO_4_ 0.02 M, 60 A/m^2^, 5 h)	advanced water treatment for MP-contaminated water	[54]
electrocoagulation (EC) using Fe and Al electrodes	coagulation and flocculation of MPs through electrochemical reactions	C	achieves 100% removal under optimal conditions; effective at pH 10 with 30 A/m^2^ current density; suitable for industrial wastewater treatment	performance dependent on current density and pH; requires optimization for large-scale applications	100% MP removal at pH 10, 30 A/m^2^ in 60 min	textile industry wastewater treatment	[55]
electrochemical technologies (EC, electro-adsorption, electro-oxidation, electro-reduction)	various electrochemical reactions for detection and removal of MP/NPs	C	multiple electrochemical methods available for both detection and removal; environmentally friendly and controllable; high removal efficiency depending on method and parameters	performance depends on electrode materials and operational conditions; requires further optimization for large-scale applications	high efficiency depending on technique (e.g., EC, electro-oxidation)	laboratory detection and industrial water treatment	[56]
microbial electrolysis cells for MPs/NP removal	biofilm-based microbial electrochemical degradation and energy recovery	B	simultaneous wastewater treatment and energy recovery; potential defense mechanism via EPS secretion; novel approach for NP degradation	high concentrations of NPs (≥500 µg/L) negatively impact microbial communities; reduced efficiency due to biofilm disruption	≈43% reduction in current density and ≈48% drop in hydrogen (H_2_) production at 500 µg/L PsNPs	bioelectrochemical systems, wastewater treatment, and energy recovery applications	[57]
machine learning & hyperspectral imaging (SVM model)	identification and quantification of MPs in rice through spectral analysis	P	high accuracy in MP identification (>94.44%); non-destructive and rapid detection; useful for assessing MP release during packaging and transportation	limited to solid food matrices like rice; may require calibration for different food products	>94.44% accuracy in identifying MPs in packaged rice	food safety, packaging analysis, and quality control	[58]
homoporous PVDF membrane separation	filtration using homoporous poly(vinylidene fluoride) membranes with high surface porosity	P	high rejection rate (>97%) for 500 nm polystyrene MPs; excellent water flux (662 L m^-2^ h^-1^ bar^-1^); simple and scalable membrane fabrication process	limited to MP sizes ≥500 nm; potential membrane fouling over prolonged use	>97% removal of 500 nm PS MPs	water treatment, MP separation in aquatic environments	[59]
EC with magnetic Fe_3_O_4_ Floc formation	coagulation and magnetic separation of MPs, with potential reuse of Fe_3_O_4_ in energy materials	C	high removal efficiency (98.4%) for PE MPs; magnetic separation enables easy recovery; recyclable Fe_3_O_4_ for lithium-ion battery applications	requires additional steps for Fe_3_O_4_ recycling; may involve complex treatment for photodegradation	98.4% removal efficiency for PE MPs	wastewater treatment and recycling for energy material applications	[60]
UV/H_2_O_2_ AOP	oxidative degradation of MP fibres using UVC irradiation and hydrogen peroxide	C	significant mass loss of MPs (52.7%) with a half-life of 45.3 h; effective surface degradation (pits, cracks); complete COD removal in 3 h	requires high doses of H_2_O_2_ (500 mg/L) and prolonged exposure to UVC (48 h); limited to photoactive treatment environments	52.7% MP mass loss, 15.2% MP degradation in hospital laundry wastewater	laundry wastewater treatment and MP degradation in industrial effluents	[61]
2D MOF@C@FeO nanopillared structures	adsorption and magnetic separation of MPs and dissolved pollutants	C	high removal efficiency (~100%) for MPs in 60 min; dual functionality for both solid and dissolved pollutants; reusable for up to 6 cycles with 90% efficiency	potential complexity in synthesis of 2D MOF@C@FeO structures; requires magnetic separation infrastructure	100% MP removal in 60 min 90% efficiency after 6 adsorption cycles	industrial and domestic wastewater treatment systems	[62]
SDS-assisted electrochemical advanced oxidation process (EAOP) with BDD anode	enhanced degradation of PS MPs using SDS and persulfate generation	C	1.35–2.29 times higher degradation rate compared to BDD electrolysis alone; effective alkyl-cleavage and oxidation of MPs; low surfactant cost (<0.05% of operating costs)	prolonged treatment time (72 h); requires precise SDS dosing strategy for optimal performance	significant improvement in MP degradation rate	AOPs in water treatment for MP removal	[63]
EC and ultrafiltration (UF) combination	coagulation followed by membrane filtration for MP recovery	C P	high recovery efficiency for MPs (85%); simultaneous removal of COD (85%), TSS (97.66%), TDS (42.74%), colour (99.74%), and turbidity (99.66%); rapid treatment time (2 min)	moderate efficiency for MP recovery compared to other methods; requires specific operational conditions (pH 7.5, NaCl 8 g)	85% MP recovery, excellent pollutant removal performance	wastewater treatment in plastic packaging industries, particularly in Vietnam	[64]
interpenetrating bipolar plate electrocoagulation (IBPE) reactor	simultaneous removal of MPs and heavy metals through EC	C	high removal efficiency for MPs (97.5%) and heavy metals (95.16%); simultaneous pollutant removal; clean technology for WWTP effluent treatment	operational cost of $0.91/L may limit large-scale use; requires precise control of current density, pH, and reaction time	97.5% MP removal, 95.16% heavy metal removal	secondary effluent treatment in WWTPs	[65]
EC with aluminum anode	flocculation and charge neutralization for MP removal	C	high removal efficiency: 93.2% for PE, 91.7% for PMMA, 98.2% for CA, and 98.4% for PP; effective across a broad pH range (3–10); works better with fiber MPs than granular MPs	requires precise control of electrolyte concentration and applied voltage (optimal: 0.05 M, 10 V); higher operational costs at non-optimal conditions	82–98.4% removal efficiency depending on MP type	municipal and industrial wastewater treatment systems	[66]
dissolved air flotation (DAF) with Al- and Fe-based coagulants	coagulation and flotation of MPs using air bubbles	C P	high PE MP removal efficiency: 96.10% (Al-based) and 70.56% (Fe-based); effective as a tertiary treatment in WWTPs; allows optimization through statistical modeling	lower efficiency with Fe-based coagulants compared to Al-based; requires precise control of pressure, pH, dosage, and flow rate	96.10% PE removal with Al-based coagulant; 70.56% with Fe-based coagulant	greywater treatment and advanced wastewater treatment in WWTPs	[67]
municipal sewage treatment plants (STPs)	multi-stage treatment including physical, chemical, and biological processes	P C B	high overall removal efficiency for MPs (>95%); capable of reducing MPs in reclaimed water to 0.59 ± 0.22 items/L; handles large volumes of wastewater	microfibers (average size: 1110.72 ± 862.95 µm) remain predominant in effluents; MPs make up 14.08% of total MPs released	>95% removal efficiency for a wide range of MP types including PET, PS, and PP	municipal wastewater treatment and water reclamation processes	[68]

^a^Type: B – biological; C – chemical; P – physical.

These technologies are indicative of the multidimensional strategies being pursued for MP remediation, which strike a balance between efficiency, operational feasibility, and environmental sustainability. While physical and chemical methods are dominant, the use of advanced technologies such as electrochemical systems, machine-learning-based sensing, and bioelectrochemical technology is a significant shift towards more precise and responsive MP management systems.

#### 5.2 Potential for bioremediation in cold environments

Psychrophiles, or coldness-adapted microbes, are essential for bioremediation under the adverse conditions of high-altitude Himalayan lakes. These microbes break down polymers like PE and PS by producing enzymes that are active below freezing temperatures [69–70]. For example, it has been discovered that certain *Pseudomonas* species efficiently break down plastics in cold climates, providing a viable solution to the problem of MP contamination in alpine ecosystems [46]. Tiwari et al. found that psychrophilic lipases and esterases found in marine environments degrade MPs in low-temperature experiments, lending credibility to their use in Himalayan habitats [42]. The ecological significance of cryophilic fungal species has been highlighted by studies showing their capacity to break down PET [46].

Biofilms can accelerate the breakdown process under cold conditions by generating enzymatically active microenvironments [71]. According to Dhiman et al., these microbial colonies stick to the surfaces of MPs and release enzymes that break down synthetic polymers into smaller, biodegradable particles [72]. Ojha et al. found that adding nutritional supplements sped up biofilm production in lab trials, suggesting that this strategy might be useful in nutrient-deficient lakes in the Himalayas [47]. The synergistic effects of hybrid biofilms that include psychrophilic bacterial and fungal species are being investigated in order to increase degrading efficiency [44].

There are several obstacles to bioremediation in alpine environments. It takes longer for plastic to degrade effectively at low temperatures because enzyme responses and microbial metabolisms are slowed down [73]. Himalayan lakes are also oligotrophic, which means they do not have a lot of nutrients. This can make it difficult for microbes to grow and for biofilm to form. To solve these problems, we might need new ideas like bioreactors designed for cold places or synthetic biology methods to improve the abilities of microbes already present [47].

#### 5.3 Emerging sustainable approaches

**5.3.1 Use of biofilms.** Biofilms have attracted a lot of interest as a remediation method for MPs because of their ecological and biological flexibility. Biofilms are groups of microbes that form a protective covering made of extracellular polymeric substances (EPSs). These EPSs are made by the microbes themselves and stick to surfaces, such as MPs. The enzymes secreted by these microbial colonies have the ability to degrade synthetic polymers into smaller, biodegradable components. Tiwari et al. have recently demonstrated the effectiveness of biofilms formed by engineered microbial consortia in expediting enzymatic degradation in response to specific environmental conditions, such as low temperatures [42].

Biofilms are very beneficial in habitats such as Himalayan lakes. Biofilm-based remediation techniques are environmentally friendly, in contrast to chemical or physical remediation techniques that could disrupt local biodiversity. For example, even in oligotrophic environments, psychrophilic bacteria in biofilms may flourish at freezing temperatures and aid in the degradation of MPs. Studies have demonstrated that electrostatically modified biofilms enhance adherence to moving water systems, rendering them a viable option for large-scale applications [43]. Biofilms along with other methods, like adding nutrients or immobilized bioreactors, have been used in field trials to show that MPs can be permanently removed without causing secondary pollution [72].

**5.3.2 Nanotechnology for degradation.** Nanotechnology offers a novel method for the accurate and effective breakdown of MPs. Under UV light, nanomaterials such as ZnO and TiO_2_ work as photocatalysts to break down MPs into CO_2_ and water, which are safe for the environment. Photocatalysis produces reactive oxygen species (ROS), which attack polymer chains and cause oxidative cleavage. Studies have shown that, under ideal circumstances, this process is quite successful in degrading polymers such as PS [74].

Bionanomaterials, which are composite systems that combine nanotechnology with biological components (e.g., enzymes), are emerging as environmentally benign solutions in addition to independent nanoparticles. Enzyme–nanoparticle conjugates can target certain plastic polymers, enabling more rapid and targeted breakdown. Iron oxide nanoparticles have been shown to break down PS in both UV and natural light, which suggests they could be used in a range of environmental conditions [44]. Another prospective application is the integration of nanomaterials into filtration membranes, which results in the development of hybrid systems that are capable of simultaneously capturing and degrading MPs. These integrated methods are especially beneficial for isolated ecosystems, such as Himalayan lakes, where minimum ecological disturbance and resource efficiency are crucial [46].

Recent studies, for example, by Ojha et al. suggest that integrating degrading enzymes from biofilms with nanomaterial carries can enhance MP degradation under cold, UV-rich conditions [47]. Such synergies offer sustainable MP removal in remote high-altitude lakes. Future work should explore their performance under freeze–thaw cycles, low sunlight penetration, and high-altitude pH variability.

### 6 Nanomaterial applications in microplastic remediation

#### 6.1 Role of photocatalysis in degradation

Photocatalysis uses nanomaterials to break down MPs by converting light energy, often UV or visible light, into ROS. These ROS, which include superoxide anions and hydroxyl radicals, attack the polymer chains of MPs. They either break the chains into smaller pieces or mineralize them into CO_2_ and water. A lot of research is being done on the photocatalytic properties of nanomaterials like TiO_2_, graphene oxide (GO), and ZnO. An excellent material for breaking down MPs in water systems is TiO_2_, which is known for being stable, effective, and able to produce ROS when exposed to UV light [43].

The effectiveness of TiO_2_ has been improved recently by doping it with nonmetals like sulfur and nitrogen or combining it with carbon-based compounds like graphene. According to Xiao et al., these changes make TiO_2_ photocatalysts more effective in natural sunlight by letting more visible light pass through them [75]. ZnO has demonstrated potential as a result of its compatibility with UV light and high oxidative potential. Its nanostructured forms, such as nanorods and nanowires, provide more surface area, allowing for better interaction with MPs [76]. The incorporation of GO in composite materials not only improves photocatalytic activity, but it also serves a second purpose by adsorbing MPs prior to degradation.

The utilization of photocatalysts that have been engineered with defects is an emerging area. The defects in the crystal structure of nanomaterials trap light energy and enhance the production of ROS. According to Kim and Youn, these developments are essential to address the robustness and effectiveness of photocatalysts in a variety of environmental conditions [77].

#### 6.2 Case studies of nanomaterial efficiency

The effectiveness of nanomaterials in the remediation of MPs has been confirmed in numerous experimental studies and real-world practical applications. Jeyaraj et al. found that after 12 h of UV irradiation, TiO_2_ nanoparticles degraded PP MPs with an efficiency of more than 80% [76]. This demonstrates their capacity to quickly clean up waterways tainted by plastic. Researchers have also demonstrated that the combination of adsorption and photocatalysis enhances the degradation of PE and PET in GO-based composites. According to Uoginte et al., GO serves as a support matrix for these composites, stabilizing the photocatalyst and enhancing its interaction with MPs [78].

Another novel strategy is the use of multifunctional nanomaterials, such as ZnO–CuO composites, which have both antibacterial and strong photocatalytic activity. These substances provide long-term performance in natural water systems by not only breaking down MPs, but also preventing the development of biofilms [79]. The use of TiO_2_ nanomaterials doped with Pt nanoparticles has also been investigated in case studies; these materials showed quicker breakdown rates for persistent PET MPs, highlighting the possibility of catalytically improved remediation techniques [80].

Nanomaterials such as ZnO have accomplished the removal of MPs from treated effluents in field tests conducted in wastewater treatment facilities. These tests were conducted under natural light and showed encouraging results. According to Goh et al., these results are especially important for expanding MP remediation technology to industrial applications [43]. Tables 4–6 presents a comprehensive comparison of various nanomaterials explored for MP remediation, detailing their synthesis methods, target pollutants, removal mechanisms, efficiency, and experimental conditions.

**Table 4:** Studies on adsorption-based nanomaterials for MP remediation.

Nanomaterial	Synthesis method	Target pollutants	Removal mechanism	Experimental conditions	Efficiency/ reusability	Ref.

sulfidised nano-zerovalent iron biochar	one-pot synthesis using sugarcane bagasse-derived biochar	amine modified NPs (AM100); sulfate modified NPs (SM100); malachite green (MG); alizarin yellow R (AY)	electrostatic attraction; dye degradation; Complexation; π–π interactions	pH: environmentally relevant conditions; instantaneous removal in <15 min	>85% 6 cycles	[81]
Fe and Al-modified chitosan (Fe-CHI and Al-CHI) ionotropic beads	modification of CHI with Fe and Al salts to form ionotropic beads	PET	adsorption via metal-ion interactions; pH-dependent protonation/deprotonation	optimal at low pH; reduced efficiency with increasing temperature	>70% 3 cycles	[82]
surface-modified nano Fe_3_O_4_ (–COOH, –NH_2_, –OH)	functionalisation of Fe_3_O_4_ NPs with carboxyl, amine and hydroxyl groups	MPs: PE nutrients: NH_4_^+^–N and PO_4_^3^–P	electrostatic adsorption; hydrogen bonding; ion exchange	adsorption capacity: NH_4_^+^–N (18.45 mg/g), PO_4_^3−^–P (30.04 mg/g), PE (1611 mg/g)	62% (in freshwater), 70% (in seawater) –	[83]
aluminum chloride (AlCl_3_) coagulant	coagulation and sedimentation process using AlCl_3_ as a coagulant	carboxyl-modified PS NPs (PS–COOH, 50 nm)	electrostatic adsorption; intermolecular interactions; surface layer compression	optimal dose: 10 mg/L AlCl_3_; pH: 3.5 to 8.5; temp.: 23 °C	96.6% (removal of 50 mg/L PS-COOH), 90.2% (removal in real surface water) –	[84]

**Table 5:** Studies on photocatalytic nanomaterials for MP remediation.

Nanomaterial	Synthesis method	Target pollutants	Removal mechanism	Experimental conditions	Efficiency/ reusability	Ref.

GO and nitrogen-doped TiO_2_ (N-TiO_2_) composites (GT13, GT11, GT31)	composite synthesis in varying weight ratios of GO:N-TiO_2_	polyvinyl chloride (PVC)	photocatalysis; adsorption under dark and light conditions	work across all pH ranges (4, 7, 10); high thermal stability	>95% –	[85]
silver-doped TiO_2_ (Ag/TiO_2_) photocatalysts (AT1.5)	photo-assisted deposition method with 1.5% Ag loading	polyamide 66 (PA66)	photocatalytic degradation; enhanced light absorption and electron–hole pair separation	UVA irradiation for 4 h; optimal catalyst-to-PA66 ratio: 3:1	58.9% (in 4 h) 100% (at optimal ratio) –	[86]
high-pressure orthorhombic phase TiO_2_	high-pressure torsion (HPT) method	PET	photoreforming; catalytic degradation and H_2_ production	light exposure to generate formic acid, terephthalate, glycolic acid, and acetic acid	high efficiency –	[87]
ZnO NPs	photocatalyst under sunlight exposure	PP	photocatalytic degradation; formation of free radicals	sunlight as the light source; UV–vis DRS and EDS analysis showed polymer chain disintegration	high –	[79]
molybdenum trioxide (MoO_3_) nanoflakes, nanobelts, and MoO_3_/ single-walled carbon nanotube (SWCNT) nanocomposites	synthesis of MoO_3_-based photocatalysts with SWCNT composites	PS NPs	photooxidative degradation under UV light; reduction in particle size and morphological transformation	UV irradiation for 24 h; PS NPs size reduced from 220 to 178 nm	>19% (with MoO_3_ nanoflakes) –	[88]
indium oxide-rGO (In_2_O_3_-rGO) nanocomposite	metal oxide nanocomposite synthesis for photocatalysis	low density polyethylene (LDPE)	photocatalytic degradation under visible light; surface morphology transformation and chemical bond cleavage	visible light exposure for up to 50 h	99.47% –	[89]
BiOCl-ZrO_2_ nanocomposite	solution casting method to blend BiOCl and ZrO_2_ into LDPE film	LDPE	photocatalytic degradation under visible light; Type II heterojunction mechanism	visible light irradiation for 24 days at room temperature	48.67% –	[90]
porous hybrid nanocomposite (HNCP) with rGO	fabrication of porous hybrid nanocomposite with rGO to enhance surface area and adsorption capacity	PET	photocatalytic degradation under light; adsorption via high surface area and pore interaction	–	64% (photocatalytic) 31% (adsorption) –	[91]
Ru-incorporated g-C_3_N_4_ (Ru-gCN) nanocomposite	blending of Ru-gCN with LDPE	LDPE	photocatalytic degradation via heterojunction formation; Norrish Type I and II mechanisms	light irradiation for 24 h; optimal pH 3; temp.: 0, 50 and 70 °C	66.04% (0 °C) 74.51% (50 °C) 69.64% (70 °C) –	[92]
TiO_2_/ZnO hybrid nanoparticles with humic substances	solvothermal synthesis using agrifood biomass-derived humic substances	polylactic acid (PLA)	photocatalytic degradation under UVA and solar light; antimicrobial activity via ROS generation	–	15–23% (carbonyl index reduction) –	[93]
CuMgAlTi-R400 quaternary layered double hydroxide composite	fabrication of a multi-metal layered double hydroxide for photocatalytic applications	- PS - PE	photocatalytic degradation under visible light; reduction of particle size and formation of hydroxyl and carbonyl intermediates	visible light irradiation: 300 h	54.2% (PS) –	[94]
GO-ZnO nanocomposite	synthesis of GO-ZnO photocatalyst with an average ZnO crystallite size of 16.43 nm	LDPE	photocatalytic degradation under UV light; interaction of pH, temp., and catalyst dosage	- pH: 9.66 - temp.: 30 °C catalyst dosage: 1500 ppm; photodegradation: 2 h	39.47% (max. MP mass loss) –	[95]
Fe- and Ag-modified TiO_2_ nanotubes	anodization of Ti plates in glycerol-based electrolyte Fe/Ag incorporation via successive ionic layer adsorption and reaction (SILAR)	PE	photocatalytic degradation under UVC light; bactericidal effect through ROS generation	90 min photoreactor test	18% (weight loss) –	[96]
Ag/TiO_2_ catalyst (3% Ag)	photoassisted deposition (PAD) method with 1%, 3%, and 5% Ag doping	PE	photocatalytic degradation under UV light; enhanced microbial disinfection via ROS	2000 rpm stirring; UV irradiation for 4 h	81% –	[97]
Fe-doped ZnO (Fe-ZnO) NPs	green synthesis using Hibiscus rosa-sinensis leaf extract	LDPE	photocatalytic degradation under sunlight; generation of ROS for antimicrobial action	sunlight exposure	high –	[98]

**Table 6:** Studies on magnetic and hybrid nanomaterials for MP remediation.

Nanomaterial	Synthesis method	Target pollutants	Removal mechanism	Experimental conditions	Efficiency/ reusability	Ref.

TiO_2_ and CuO-modified polyvinylidene fluoride (PVDF) nanofibrous composite membranes	needle-free electrospinning of PVDF nanofibers with lamination and modification using alkaline treatment, biosurfactant, TiO_2_, and CuO particles	MPs (0.5 µm); oil in oil-water emulsions	physical separation; antifouling through TiO_2_ and CuO particles	BS-modified membranes: >9000 L·m^−2^·h^−1^·bar^−1^ permeability for MP separation; TiO_2_-modified membranes: high oil rejection (≈95%)	99.99% (MP) 95.30% (oil) –	[99]
fly ash-loaded magnetic nanoparticles (FAMNPs)	synthesis of magnetic nano-adsorbent using fly ash	PS	adsorptive uptake via magnetic separation	pH 8.5; FAMNPs dose: 0.03 g; adsorption capacity: 29.12 mg/g	high adsorption capacity –	[100]
magnetic composite NPs (FNP)	synthesis of Fe_3_O_4_ magnetic cores with eco-friendly coatings (silicon dioxide, CHI, gelatine)	PET (5–30 µm)	magnetic sedimentation; heteroaggregation of FNP and PET	magnetic field strength: 0.44 T; optimal dose: 0.002 g/L; sedimentation time: 0.5 h	>98% –	[101]
magnetic ZIF-8 NPs (Nano-Fe@ZIF-8)	synthesis of magnetic MOFs with Fe and Zn (II) components	PET	magnetic aggregation and retrieval; catalytic depolymerisation	depolymerisation with ethylene glycol at 180 °C; rapid process: 30 min	high –	[102]
nickel/reduced graphene oxide (Ni/rGO) nanocomposite	magnetisation of rGO using Ni NPs	PS	adsorption via hydrophobic interactions; magnetic separation	maximum adsorption capacity: 1250 mg/g; follows Langmuir isotherm and pseudo second-order kinetics	high 3 cycles	[103]
hydrogen titanate nanotubes (HTNT@ZIF-67) nanocomposite	combination of HTNT with zeolite imidazolate frameworks (ZIF-67) at ambient temperature	MPs from personal care products (PCPs) and cosmetic products	adsorption; catalytic oxidation with H_2_O_2_	–	99% (adsorption) 97% (catalytic oxidation) 6 cycles	[104]
Fe_3_O_4_@PDA (polymerised dopamine-coated Fe_3_O_4_ NPs)	coating of mesoporous Fe_3_O_4_ NPs with adhesive PDA to mimic coral reef structures	MPs	adsorption through hydrogen bonding, π–π stacking, and hydrophobic interactions	–	98.5% –	[105]
laundry filter system with Fe_3_O_4_ and CaCO_3_	incorporation of magnetic (Fe_3_O_4_) and carbonate (CaCO_3_) particles into filter design	MP	simultaneous removal of hydrophobic and hydrophilic MPs; adsorption and filtration	–	78.3–89% –	[106]
*n*-butylamine modulated magnetic ZIF-8 (nano-Fe@ZIF-8)	synthesis of magnetic ZIF with *n*-butylamine in water at room temp	PS (1.1 µm)	adsorption and magnetic separation; fast removal of MPs and phenolic compounds	dosage: 20 mg	≥98% –	[107]
superhydrophobic magnetic adsorbents (Fe_3_O_4_@Cn)	liquid phase deposition of Fe_3_O_4_ with saturated fatty acids (C12, C14, C16, C18)	PS	adsorption through electrostatic and chemical bonding interactions; magnetic separation	–	92.89% (Fe_3_O_4_@C12) 809.29 mg/g (adsorption capacity) –	[108]
magnetic effervescent tablet with deep eutectic solvent (DES)	formulation of effervescent tablet containing DES, Fe_3_O_4_ magnetic NPs, sodium carbonate, and tartaric acid	PS	combined extraction and adsorption through DES and magnetic NPs; effervescence aids in rapid dispersion and sorption	optimal conditions: 94.0 ± 0.8% extraction efficiency; detection via fluorescence-assisted method	94% –	[109]
multiwalled carbon nanotube (MWCNT) membrane	fabrication of lightweight, freestanding, and flexible MWCNT membranes	MPs	physical sieving through narrow pore size (≈16 nm); hydrophobic surface for self-cleaning	contact angle: ≈148°; high porosity: 56%; pressure drop: ≈139.7 Pa	>99% –	[110]
magnetic carbon nanotubes (M-CNTs)	synthesis of M-CNTs for adsorption and magnetic separation	PE; PET; PA	adsorption of MPs onto M-CNTs; magnetic separation for removal	M-CNTs: 5 g/L; time: 300 min; max. adsorption capacity: 1650 mg/g (PE), 1400 mg/g (PET), 1100 mg/g (PA)	100% (MP removal from solution) ≈80% (MP removal after 4 reuse cycles) 4 cycles	[111]

To improve clarity and compare functional mechanisms, the nanomaterials are grouped based on their primary MP removal strategy, that is, adsorption, photocatalysis, and magnetic/hybrid systems. Tables 4–6 highlight that adsorption and photocatalysis are the most prevalent mechanisms in nanomaterials to eliminate MPs, where metal oxides and carbon materials are particularly effective. Surface modification and environmental factors are crucial in controlling removal.

#### 6.3 Tailoring nanomaterials for low-temperature, high-UV environments

The Himalayan lakes and other high-altitude environments provide specific challenges for MP removal because of their low temperatures, intense UV radiation, and scarcity of organic materials. It is imperative that nanomaterials are engineered to maintain photocatalytic efficiency under cold conditions while simultaneously utilizing the abundant UV radiation [75]. It has been demonstrated that the light absorption of ZnO and TiO_2_ is improved by defect engineering, such as the introduction of oxygen vacancies. According to Kim and Youn, these defects trap light energy, which lowers charge carrier recombination rates and increases ROS generation, even below freezing temperatures. It has also been suggested that hybrid nanomaterials, which include photocatalysts and thermal insulators, can minimize the impacts of temperature during remediation while maintaining optimum activity [77].

GO–metal oxide composites are another promising approach. GO may be combined with TiO_2_ or ZnO to create materials that have both adsorption and photocatalytic properties, which improves their ability to absorb and break down MPs. They are perfect for harsh and isolated environments because of their resilience and flexibility [79]. The development of scalable technologies for producing nanomaterials is essential for their practical application. Green chemistry methods, such as plant-based precursors or bioinspired templates, have been successfully used to synthesize nanomaterials with high photocatalytic efficiency and minimal environmental effect [77].

Despite their high efficiency, nanotechnology-based remediation solutions may face deployment challenges in remote Himalayan regions due to high initial cost, maintenance needs, and lack of infrastructure. Scalable, low-cost nanocomposites or hybrid bionanotechnology systems adapted for cold climates represent a promising direction for future trials.

### 7 Ecotoxicological impact and risk assessment

MP pollution of high-altitude ecosystems is a new issue with significant ecological and health effects. Because of low temperature, high levels of UV irradiation, and slow biodegradation rates, MPs remain long enough in aquatic environments, boosting their ecotoxicological impact [4]. This section covers the consequences of MPs for aquatic biodiversity, bioaccumulation within the food chain, additive toxicity of plastic, and likely long-term health effect on humans and ecosystems.

#### 7.1 Bioaccumulation of microplastics in the Himalayan food web

MPs are consumed by numerous aquatic animals, such as plankton, benthic invertebrates, and fish, resulting in bioaccumulation and biomagnification in the Himalayan food web [112]. MP ingestion has been documented in freshwater fish populations of high-altitude lakes, where particles accumulate in the gut and gill tissues and interfere with normal physiological functions [113]. The low molecular weight of MPs, and particularly nanoparticles (NPs), allows them to easily translocate into organs and tissues resulting in inflammation, oxidative stress, and metabolic disturbance in aquatic life [114].

MPs also act as carriers of hydrophobic organic pollutants such as pesticides, heavy metals, and persistent organic pollutants, which are more harmful when ingested [23]. Trophic transfer issues are generated by the bioaccumulation of MPs in fish, with birds and mammals being exposed to tainted prey, which might have implications at the ecosystem level. The occurrence of MPs in freshwater fauna of the Himalayas also implies that native human populations dependent upon fish as food could be endangered by MP ingestion.

#### 7.2 Toxicity from plastic additives and degradation byproducts

MPs come from various sources and are responsible for environmental and human health risks. First, plastics are manufactured with chemical additives such as phthalates, bisphenol A (BPA), and brominated flame retardants, which are endocrine disruptors and carcinogens [115–116]. Second, MPs have a large surface-area-to-volume ratio that enables them to adsorb persistent organic pollutants, polycyclic aromatic hydrocarbons, and heavy metals, such as Cd, Pb, and Hg from bodies of water [5,117]. Third, photodegradation and oxidative weathering of MPs upon UV light and temperature fluctuations release toxic byproducts and nanoplastics, which are more bioavailable and more easily taken up by cells [118–119].

These collective mechanisms of toxicity are a threat to freshwater biodiversity and human health. Their activity is particularly vital to individuals relying on such water for drinking, agriculture, and fisheries. Their occurrence and persistence are therefore of utmost significance to Sustainable Development Goal 3 (SDG 3, Good Health and Well-Being), SDG 6 (Clean Water and Sanitation), SDG 12 (Responsible Consumption and Production), and SDG 14 (Life Below Water). Apart from their physical structure, MPs are also significantly hazardous through the additives and byproducts that they release into the environment [120–121].

#### 7.3 Long-term implications for ecosystem and human health

Long-term exposure to MPs can potentially alter fundamental ecological processes such as nutrient cycling and primary production through the influence on keystone organisms in the ecosystem [11]. Microbial community disruption due to MP pollution has been observed, which can disrupt the balance of prevalent nutrient conversions in freshwater ecosystems [113]. From a public health perspective, populations residing around Himalayan lakes and dependent on these freshwater basins for agriculture, drinking water, and fishery resources are more likely to be exposed to MPs. Although comprehensive data on MP bioaccumulation in Himalayan populations are currently lacking, the detection of MPs in Himalayan spring water and fish highlights potential exposure. This gap underscores the urgent need for region-specific health monitoring and food safety assessments. Recent studies report the identification of MPs in bottled and mountain spring-collected tap water samples and their bioaccumulation in the human body upon ingestion as an emerging issue [10].

With increasing evidence of MP pollution in remote, high-altitude ecosystems, there is a pressing need to limit their impacts. Establishment of robust surveillance regimes, policy measures, and ecofriendly waste disposal methods in the Himalayas is required. Future research needs to include the transport and fate of MPs in extreme ecosystems, their synergistic impacts with other pollutants, and their long-term effects on biodiversity and human health.

### 8 Policy frameworks and management strategies for MP pollution

#### 8.1 Current policies addressing MP pollution

MP pollution has emerged into the limelight internationally and has led to many policies and regulations addressing its effects. At the international scale, the United Nations Environment Programme (UNEP) has demonstrated significant initiative in the form of deliberations of a Global Plastic Treaty with an aim to constitute legally binding plastic reduction instruments along with MP reduction. Consequently, the European Union (EU) also deliberately limited incorporated MPs in the EU Microplastic Restriction Proposal of the REACH regulation of cosmetics, cleaning products, and agricultural products in a bid not to release MPs into the environment [122].

National governments have also implemented policies to avoid MP pollution. The United States enacted the Microbead-Free Waters Act (2015), which banned microbeads from plastic in personal care products. California and other states have more recently enacted even more stringent regulations for the tracking of MPs in drinking water. In India, the Plastic Waste Management Rules (amended 2016 and 2021) prohibit microbeads in cosmetics and impose extended producer responsibility (EPR) on producers of plastic to enable recycling and green disposal. China, in turn, has initiated its plastic pollution control action plan, and it put special emphasis on reducing MPs through strict prohibitions on some plastic imports and improved waste management practices [123]. The key national and regional policy frameworks aimed at mitigating MP pollution are discussed in Supporting Information File 1, Table S1.

However, these advancements are not well followed up with the implementation of the policies, especially in the global south. Standardized tests and monitoring procedures for air-, water-, and food-borne MPs do not exist in the majority of countries. Furthermore, opposition from industries to phasing out synthetic MPs and lack of sufficient public awareness slow down the process. Upcoming policies must address more monitoring, industry compliance, and investment in sustainable alternatives to plastics [124].

#### 8.2 Community-driven approaches in the Himalayan context

The Himalayas experience MP pollution from tourism, rampant waste dumping, and plastic waste transport as a result of glacial melts [125]. Locally driven interventions have been helpful steps in the fight against such issues to ensure and hold back plastic pollution. These successful citizen-led initiatives among others are single-use plastic prohibitions at the local government level and waste management programs. Sikkim took the lead as the first Indian state to prohibit single-use plastics back in 1998, and later Ladakh, Himachal Pradesh, and Bhutan followed suit [126]. The prohibitions have been implemented through joint efforts of citizen volunteers, NGOs, and local administrations.

Citizen science programs in recent times have played a vital role in monitoring and alleviating MP pollution in Himalayan rivers. WWF, Eco Himal, and The Himalayan Cleanup are organizations that have mobilized local communities and school children to collect and analyze water samples regarding MP contamination [127]. In Nepal, zero-waste trekking initiatives at Everest Base Camp and Annapurna Circuit encourage hikers to collect waste, reducing the plastic waste in these wilderness areas [128–129].

Apart from this, novel plastic exchange initiatives have been initiated in far-flung Himalayan villages. By these programs, citizens can trade plastic waste collected by them for essential items such as rice, vegetables, or school supplies, encouraging waste management practice among citizens. By promoting awareness campaigns for plastic-free religious pilgrimages such as the Hemis Festival in Ladakh, environmentally friendly practices among pilgrims and tourists have been encouraged [130]. With these initiatives, however, come challenges in collection infrastructure, enforcement of policy, and funding local efforts. For these community-driven strategies to scale up, however, more government support, inter-border cooperation, and coordination with formal waste management systems are necessary.

#### 8.3 Suggestion for monitoring and regulations

The increasing presence of MPs in the environment calls for effective monitoring systems and stringent regulatory systems to mitigate their impacts. Governments and international agencies must give the highest priority to standardized detection methods, legislative measures, industry accountability, and public awareness to combat this issue effectively.

First, detection and monitoring systems have to be boosted in order to build a baseline of MP pollution control. Standard protocols created by international organizations can monitor MPs in a variety of environmental matrices. Analysis methods such as Raman spectroscopy, FTIR, and pyrolysis-GC/MS have been accepted as highly effective to trace the origins of MPs and compositions [131]. Public engagement in monitoring programs, such as citizen science initiatives, plays a crucial role in data collection and raising awareness [132].

Regulatory systems play a key part in managing MPs pollution. Bans of microbeads added intentionally have been put into practice by some countries, such as Canada and the UK, where microbeads are banned in cosmetics [133]. The EU has placed restrictions under REACH regulation to restrict commercial use of MPs [134]. France has taken further steps by mandating that new washing machines be equipped with microfiber filters by 2025 to reduce microfiber emissions [132]. In addition, the United States has taken actions such as the “Save Our Seas 2.0 Act”, which provides grants to enhance wastewater treatment infrastructure and reduce MPs contamination [135].

Since wastewater treatment plants (WWTPs) are significant contributors to MP pollution, upgrading filtration technology is crucial. Germany and Switzerland have also enhanced their WWTPs to add more advanced membrane bioreactors and electrocoagulation systems to minimize MP discharge [136]. Implementation of microfiber filters for laundry, already taken up in France and debated in Australia, additionally limits MP discharge from clothes [137]. Encouraging a circular economy model can minimize plastic waste generation at the industrial level. EPR policies have been proposed to hold manufacturers accountable for post-consumer plastic waste, as emphasized in the EU’s Circular Economy Action Plan [138]. Additionally, industries should be incentivized to develop biodegradable alternatives, such as bioplastics, to reduce dependency on conventional plastics [139].

Due to the transboundary character of MP pollution, international collaboration is necessary. The 2022 UN Global Treaty on Plastics, which was signed by 175 countries, is a major milestone toward legally binding global agreements on plastic waste management [140], including MP cross-border policy coordination and research, supported by efforts such as the global partnership on marine litter (GPML), strengthening global action against MP pollution [133]. A comprehensive solution to MP pollution must involve interdisciplinary measures that combine scientific monitoring, strict legislation, technological innovation, and cooperation from industries. Strengthened policies, enhanced purification techniques, and international cooperation will help governments to reduce MP contamination by a considerable extent, creating a safer environment for future generations.

### 9 Research gaps and future directions

Even with increasing recognition of MP pollution, there are still major research gaps regarding fate, impact, and mitigation of MPs. One such challenge is the absence of a standardized monitoring system, which causes variability in data collection between studies. More advanced spectroscopic and AI-based detection systems need to be investigated for enhanced accuracy and efficiency. Furthermore, the long-term destiny, transport, and bioaccumulation of MPs in the different compartments of the environment have not been well established, especially about how they interact with other contaminants and can potentially be transferred along food webs. Human health effects of MPs are another important knowledge gap since toxicity mechanisms, exposure routes, and long-term risks are still not well established. It should be directed towards understanding ingestion, inhalation, and dermal exposure pathways, as well as to MP ability to act as a vector of toxic chemicals and microorganisms.

Emerging MP removal technologies and regulatory policies also need to be developed. Existing wastewater treatment facilities are not effective in completely filtering MPs, so membrane filtration, electrocoagulation, and biodegradable plastic substitutes need to be improved. While some countries have implemented bans on microbeads, comprehensive regulations addressing other sources, such as textiles and industrial waste, are still lacking. A stronger policy framework, encompassing extended producer responsibility and circular economy models, is required to constrain MP pollution. Socioeconomic impacts, including impacts on fisheries and tourism, must be better understood to improve policymaking. Future studies must take a multidisciplinary approach with the inclusion of environmental science, public health, engineering, and policy to develop holistic strategies to mitigate MP pollution and ensure long-term sustainability.

## Conclusion

This review points out that among the serious environmental problems created by tourism, atmospheric deposition, and melting of glaciers is microplastic contamination of Himalayan Lakes. The severity of climatic conditions and the need for precise analytical methods make the assessment of microplastic contamination in such remote environments difficult.

Physical technologies such as adsorption and filtration with materials such as nanomaterials and biochar can be used to remove microplastics from water. But they may need high energy input. Chemical processes such as oxidation and coagulation can degrade microplastics, but they may produce secondary pollutants. Biological processes, especially microplastic degradation aided by biofilms and microbial consortia, are challenging regarding long-term sustainability and effectiveness in cold, high-altitude environments. Nanotechnology-based treatments, especially photocatalytic degradation, are strongly effective in removing microplastics with less environmental damage. The most promising approach, balancing effectiveness with sustainability, is a blend of biological and nanotechnology-based remediation, keeping in view the eco-sensitive nature of the Himalayan lakes. For effective microplastic reduction in natural aquatic environments, future studies must be directed towards field-scale application and long-term ecological consequences of these technologies.

This review fills an essential knowledge gap by specifically examining MP pollution in high-altitude Himalayan lake ecosystems. Through the incorporation of knowledge on glacial melting, tourism pressure, and groundwater connectivity, the review adds to a regional-scale understanding of MP dynamics. In addition, the research discusses specific recommendations for monitoring and mitigation, including the application of nanotechnology-based solutions and SDG-linked governance frameworks. These individual contributions, in total, close the research gap that was identified and provide a roadmap for future research and policy intervention into cold-climate freshwater systems.

## Supporting Information

File 1 Additional table.

## Data Availability

Data sharing is not applicable as no new data was generated or analyzed in this study.
