# Supplementary material for: Microplastic pollution in Himalayan lakes: assessment, risks, and sustainable remediation strategies
Source: Beilstein J Nanotechnol. 2025 Nov 25;16:2144–67. doi: 10.3762/bjnano.16.148 (PMC12667730; doi:10.3762/bjnano.16.148)
Supplement: File 1 — Additional table. [file Beilstein_J_Nanotechnol-16-2144-s001.pdf]

BEILSTEIN JOURNAL OF NANOTECHNOLOGY

Supporting Information

for

Microplastic pollution in Himalayan lakes: assessment, risks, and sustainable remediation strategies

Sameeksha Rawat, S. M. Tauseef and Madhuben Sharma

Beilstein J. Nanotechnol. **2025,** *16,* 2144–2167. doi:10.3762/bjnano.16.148

Additional table

License and Terms: This is a supporting information file under the terms of the Creative Commons Attribution License (https://creativecommons.org/licenses/by/4.0). Please note that the reuse, redistribution and reproduction in particular requires that the author(s) and source are credited and that individual graphics may be subject to special legal provisions.
The license is subject to the *Beilstein Journal of Nanotechnology* terms and conditions: (https://www.beilstein-journals.org/bjnano/terms)

Table S1: Representative regional and national MP policy measures (modified from [1]).

Year	Country	Target	Policy name	Category	Description	Reference
2022	Canada	Microfibers	Environmental Protection Amendment Act (Microfiber Filters for Washing Machines), 2022	Regulation	The Bill amends the Environmental Protection Act to ban selling washing machines without a specified microfiber filter and imposes penalties for non-compliance.	[2]
2022	China	Microbeads	Notice on Solidly Promoting Plastic Pollution Control	Policy	Lays out responsibilities for ensuring compliance to microbead bans.	[3]
2022	Finland	General	Programme of Measures of the Finnish Marine Strategy 2022-2027	Action Plan	The MSFD program includes measures to reduce MP loads by addressing shipping, marinas, road transport, agriculture, wastewater and urban drainage, and artificial grass, abandoned glass fiber boats, and marine pollution.	[4]
2022	France	Plastic pellets, flakes and powders	Decree No. 2021-461 of 16 April 2021 on the prevention of losses of industrial plastic pellets into the environment	Regulation	Mandates regulations for industrial plastic pellet production, handling, and transport to prevent environmental MP pollution, with regular inspections by certified bodies.	[5]
2022	Spain	Microbeads; oxo-degradable plastics	Law 7/2022, of April 8, on waste and contaminated soil for a circular economy	Regulation	Prohibits the sale of certain plastic products, including oxodegradable plastic and intentionally added plastic microspheres under 5 mm, following the REACH Regulation.	[6]
2021	Australia	Microbeads; microfibers	National Plastic Plan 2021	Action Plan	A voluntary industry-led phase-out of microbeads in rinse-off personal care, cosmetics and oral hygiene products.	[7]
2021	China	Microbeads; research; monitoring; recycling; waste management	14th Five Year Plan Plastic Pollution Control Action Plan	Action Plan	Includes source reduction of plastic pollution (microbead ban), research on MP monitoring and control in water bodies, and measures for recycling and waste management.	[8]
2021	Italy	Wastewater; PS	Updated Programme of measures according to Article 13 of the MSFD:	Action Plan	Develops a supply chain for reusable fish boxes and tests prototypes for MP removal WWTPs.	[9]
2021	Norway	Rubber infill	Chapter 23A of the Regulations relating to pollution control	Regulation	In March 2021, Norway introduced regulations for the design and operation of sports pitches using loose MP infill, effective from July 1, 2021.	[10]

2021	South Korea	Microbeads	Regulation on use of micro-bead	Regulation	Ban the use of microbeads in all cleansing agents, detergents and removers manufactured in and outside of South Korea.	[11]
2021	USA	Microfibers; wastewater	Save our Seas 2.0 Act	Regulation	The act allows the US Environmental Protection Agency to fund projects aimed at reducing microfiber and MP pollution in drinking water and wastewater treatment.	[12]
2020	Argentina	Microbeads	Law27602	Regulation	Bans the production, import and sale of cosmetic products and dental hygiene products for containing intentionally added plastic microbeads.	[13]
2020	China	Microbeads	Opinions on Further Strengthening the Control of Plastic Pollution	Regulation	Prohibit the production of daily chemical products containing microbeads by the end of 2020 and in cleaning products by the end of 2022.	[14]
2020	France	Microfibers	Law No. 2020-105 of February 10, 2020 relating to the fight against waste and the circular economy	Regulation	From January 1, 2025, all new washing machines must have a plastic microfiber filter or an equivalent solution. A decree outlines implementation detail. The government was required to report to Parliament by December 31, 2022, on microfiber emissions, industry constraints and voluntary reduction measures.	[15]
2020	Thailand	Microbeads	Plastic Management Plan B.E. 2561-2573	Regulation	Ban on the use of plastic microbeads in the import, production and sale of cosmetics.	[16]
2019	Ireland	Microbeads	Microbeads (Prohibition) Act 2019	Regulation	Ban on production and sale of rinse-off cosmetics and household and cleaning products with plastic microbeads.	[17]
2019	Peru	PS; oxo-degradable plastics	Law N°30884 "Law that Regulates Single-Use Plastic and Disposable Containers or Containers"	Regulation	The law prohibits single-use plastic items, including bags, straws, and expanded PS food containers, as well as plastics with additives that cause MP contamination or contain hazardous, non-recyclable substances.	[18]
2018	Germany	Microbeads	Ban of microplastics in soaps, creams, toothpastes	Regulation	As of January 1, 2020, the law bans the sale of rinse-off cosmetic products with exfoliating or cleansing MPs, with penalties ranging from fines to production halts.	[19]
2018	Italy	Microbeads	General Budget Law 2018: Law no. 205 of 27, Art. 1, Sections 543 to 548, December 2017	Regulation	Ban on the production and marketing of rinse-off cosmetics products (including soaps, creams and toothpastes) containing MPs.	[20]

2018	Sweden	General	Regulation (2018: 496) on state subsidies to reduce emissions of microplastics to the aquatic environment	Regulation	Public support for investments in technology and measures to clean surface water from MPs and other contaminants, along with feasibility studies and preparatory measures.	[21]
2018	Sweden	Microbeads; oxo-degradable plastics	Prohibition in Certain Cases in Connection with the Handling, Import and Export of Chemical Products Ordinance (1998:944)	Regulation	Ban on the import and manufacture of microbeads in rinse-off cosmetics (2018), sale of microbeads in rinse-off cosmetics (2019), and oxo-degradable plastics (2021).	[22]
2018	The Netherlands	General	Dutch Policy Programme on Microplastics	Strategy	The programme aims to ban intentional MP additives at the European level, reduce emissions from plastic litter degradation, minimize MP release from wear and tear of products (e.g., tires, paint, clothing), and enhance research on their effects on human health.	[23]
2018	United Kingdom (Scotland)	Microbeads	The Environmental Protection (Microbeads) (Scotland) Regulations 2018	Regulation	Ban on the manufacture and supply of rinse-off PCPs containing microbeads.	[24]
2018	United Kingdom (Wales)	Microbeads	Environmental Protection (Microbeads) (Wales) Regulations 2018 (S.I. No. 760 (W. 151) of 2018)	Regulation	Ban on the manufacture and supply of rinse-off PCPs containing microbeads.	[25]
2018	USA (California)	General	SB 1422: California Safe Drinking Water Act	Policy	Decision to determine MPs in drinking water, start a monitoring project, and provide information to constituents.	[26]
2018	USA (California)	General	SB 1263: Microplastic Materials	Policy	Decision and description of how to develop a statewide MPs strategy.	[27]
2018	USA (California)	General	California Ocean Litter Prevention Strategy	Strategy	The strategy focuses on understanding the scale and impact of MPs and microfibers in marine environments and developing solutions to mitigate them. Key objectives include conducting comprehensive characterization of MPs and macro-debris (Objective 4.1) and quantifying MP pathways within watersheds while developing technological solutions for their reduction (Objective 4.2).	[28]

2017	Canada	Microbeads	Microbeads in Toiletries Regulations (SOR/2017-111)	Regulation	The regulation bans the manufacture, import, and sale of exfoliating or cleansing toiletries containing plastic microbeads, including non-prescription drugs and natural health products, with a phased ban starting January 1, 2018, and a complete ban by July 2019.	[29]
2017	China (Taiwan)	Microbeads	Restrictions on the Manufacture, Import, and Sale of Personal Care and Cosmetics Products Containing Plastic Microbeads	Regulation	Ban on import, manufacture and sale of microbeads in rinse-off cosmetics	[30]
2017	New Zealand	Microbeads	Waste Minimization (Microbeads) Regulations 2017, under section 23(1) (b) of the Waste Minimization Act 2008	Regulation	Ban on import, manufacture, and sale of wash-off products containing microbeads for exfoliation, body cleaning, abrasive cleaning, or virtual appearance, excluding medical devices or medicines	[31]
2017	South Korea	Microbeads	Regulations on safety standards for cosmetics [Annex 1] {No. 2017-114, Notice, Article 3, Dec, 29, 2017	Regulation	Ban on sale all cosmetic products containing MPs under 5 mm	[32]
2017	United Kingdom (England)	Microbeads	The Environmental Protection (Microbeads) (England) Regulations 2017	Regulation	Ban on the manufacture and supply of rinse-off PCPs containing microbeads	[33]
2016	France	Microbeads	Reclaiming Biodiversity, Nature and Landscapes Act No 2016-1087 of 8, Article 124, August 2016	Regulation	Bans the sale of rinse-off cosmetic products with plastic microbeads for exfoliation or cleaning from 2018	[34]
2015	Canada (Ontario)	Microbeads	Bill 75 Microbead Elimination and Monitoring Act (2015)	Regulation	Prohibits the manufacture and addition of microbeads in cosmetics, soaps, and similar products	[35]
2015	USA	Microbeads	Microbead-Free Waters Act	Regulation	Prohibits the sale and distribution of rinse-off cosmetics with plastic microbeads. Manufacturing banned from July 2017, interstate delivery prohibited by 2018, with a one-year delay for non-prescription drugs	[36]

2022	China	Microbeads	Notice on Solidly Promoting Plastic Pollution Control	Policy	Lays out responsibilities for ensuring compliance to microbead bans.	[3]
2021	China	Microbeads; research; monitoring; recycling; waste management	14th Five Year Plan Plastic Pollution Control Action Plan	Action Plan	Includes source reduction of plastic pollution (microbead ban), research on MP monitoring and control in water bodies, and measures for recycling and waste management.	[37]
2021	South Korea	Microbeads	Regulation on use of micro-bead	Regulation	Ban the use of microbeads in all cleansing agents, detergents and removers manufactured in and outside of South Korea.	[38]
2020	China	Microbeads	Opinions on Further Strengthening the Control of Plastic Pollution	Regulation	Prohibit the production of daily chemical products containing microbeads by the end of 2020 and in cleaning products by the end of 2022.	[39]
2017	China (Taiwan)	Microbeads	Restrictions on the Manufacture, Import, and Sale of Personal Care and Cosmetics Products Containing Plastic Microbeads	Regulation	Ban on import, manufacture and sale of microbeads in rinse-off cosmetics	[40]
2017	South Korea	Microbeads	Regulations on safety standards for cosmetics [Annex 1] {No. 2017-114, Notice, Article 3, Dec, 29, 2017	Regulation	Ban on sale all cosmetic products containing MPs under 5 mm	[41]
2022	France	Plastic pellets, flakes and powders	Decree No. 2021-461 of 16 April 2021 on the prevention of losses of industrial plastic pellets into the environment	Regulation	Mandates regulations for industrial plastic pellet production, handling, and transport to prevent environmental MP pollution, with regular inspections by certified bodies.	[42]
2020	France	Microfibers	Law No. 2020-105 of February 10, 2020 relating to the fight against waste and the circular economy	Regulation	From January 1, 2025, all new washing machines must have a plastic microfiber filter or an equivalent solution. A decree outlines implementation detail. The government was required to report to Parliament by December 31, 2022, on microfiber emissions, industry constraints and voluntary reduction measures.	[43]

2018	Germany	Microbeads	Ban of microplastics in soaps, creams, toothpastes	Regulation	As of January 1, 2020, the law bans the sale of rinse-off cosmetic products with exfoliating or cleansing MPs, with penalties ranging from fines to production halts.	[44]
2018	United Kingdom (Scotland)	Microbeads	The Environmental Protection (Microbeads) (Scotland) Regulations 2018	Regulation	Ban on the manufacture and supply of rinse-off PCPs containing microbeads.	[45]
2016	France	Microbeads	Reclaiming Biodiversity, Nature and Landscapes Act No 2016-1087 of 8, Article 124, August 2016	Regulation	Bans the sale of rinse-off cosmetic products with plastic microbeads for exfoliation or cleaning from 2018	[46]
2018	USA (California)	General	SB 1422: California Safe Drinking Water Act	Policy	Decision to determine MPs in drinking water, start a monitoring project, and provide information to constituents.	[47]
2018	USA (California)	General	SB 1263: Microplastic Materials	Policy	Decision and description of how to develop a statewide MPs strategy.	[48]
2018	USA (California)	General	California Ocean Litter Prevention Strategy	Strategy	The strategy focuses on understanding the scale and impact of MPs and microfibers in marine environments and developing solutions to mitigate them. Key objectives include conducting comprehensive characterization of MPs and macro-debris (Objective 4.1) and quantifying MP pathways within watersheds while developing technological solutions for their reduction (Objective 4.2).	[49]
2017	Canada	Microbeads	Microbeads in Toiletries Regulations (SOR/2017-111)	Regulation	The regulation bans the manufacture, import, and sale of exfoliating or cleansing toiletries containing plastic microbeads, including non-prescription drugs and natural health products, with a phased ban starting January 1, 2018, and a complete ban by July 2019.	[50]
2015	Canada (Ontario)	Microbeads	Bill 75 Microbead Elimination and Monitoring Act (2015)	Regulation	Prohibits the manufacture and addition of microbeads in cosmetics, soaps, and similar products	[51]
2021	Australia	Microbeads; microfibers	National Plastic Plan 2021	Action Plan	A voluntary industry-led phase-out of microbeads in rinse-off personal care, cosmetics and oral hygiene products.	[52]

2020	Argentina	Microbeads	Law27602	Regulation	Bans the production, import and sale of cosmetic products and dental hygiene products for containing intentionally added plastic microbeads.	[53]
2020	Thailand	Microbeads	Plastic Management Plan B.E. 2561-2573	Regulation	Ban on the use of plastic microbeads in the import, production and sale of cosmetics.	[54]

References

1. Rognerud, I.; Hurley, R.; Lusher, A.; Nerland Bråte, I. L.; Hovland Steindal, E. *Addressing microplastics in a global agreement on plastic pollution*. Nordic Council of Ministers. **2023**. doi:10.6027/temanord2022-566
2. An Act to Amend the Environmental Protection Act with Respect to Microfiber Filters for Washing Machines (2022). https://www.ola.org/en/legislative-business/bills/parliament-42/session-2/bill-102
3. Notice on Solidly Promoting Plastic Pollution Control (2020). https://www.mee.gov.cn/xxgk2018/xxgk/xxgk10/202007/t20200717_789638.html
4. https://julkaisut.valtioneuvosto.fi/bitstream/handle/10024/163705/YM_2021_31.pdf?sequence=1&isAllowed=y
5. https://www.legifrance.gouv.fr/jorf/id/JORFTEXT000043388114
6. https://www.boe.es/buscar/act.php?id=BOE-A-2022-5809
7. https://www.agriculture.gov.au/sites/default/files/documents/national-plastics-plan-2021.pdf
8. https://www.mee.gov.cn/xxgk2018/xxgk/xxgk10/202109/t20210916_945621.html
9. https://g20mpl.org/wp-content/uploads/2022/08/G20MPL-report-2022_2nd-edition_1108.pdf
10. https://www.miljodirektoratet.no/ansvarsomrader/avfall/avfallstyper/gummigranulat-fra-kunstgressbaner/
11. https://g20mpl.org/partners/republicofkorea
12. https://www.congress.gov/bill/116th-congress/senate-bill/1982/text
13. https://www.argentina.gob.ar/normativa/nacional/ley-27602-345720
14. https://www.ndrc.gov.cn/xxgk/zcfb/tz/202001/t20200119_1219275.html?code=&state=123
15. https://www.legifrance.gouv.fr/jorf/id/JORFTEXT000043956924
16. https://g20mpl.org/partners/thailand
17. https://www.irishstatutebook.ie/eli/2019/act/52/enacted/en/print
18. https://g20mpl.org/wp-content/uploads/2022/08/G20MPL-report-2022_2nd-edition_1108.pdf
19. https://g20mpl.org/wp-content/uploads/2022/08/G20MPL-report-2022_2nd-edition_1108.pdf
20. http://www.normattiva.it/eli/id/2017/12/29/17G00222/CONSOLIDATED/20221222
21. http://www.fao.org/faolex/results/details/en/c/LEX-FAOC187073

22. http://www.riksdagen.se/sv/dokument-lagar/dokument/svensk-forfattningssamling/forordning-1998944-om-forbud-mm-i-vissa-fall_sfs-1998-944
23. https://www.tweedekamer.nl/downloads/document?id=2021D25682
24. https://nicholasinstitute.duke.edu/plastics-policies/environmental-protection-microbeads-scotland-regulations-2018
25. https://nicholasinstitute.duke.edu/plastics-policies/environmental-protection-microbeads-wales-regulations-2018-si-no-760-w-151-2018
26. https://leginfo.legislature.ca.gov/faces/codes_displaySection.xhtml?sectionNum=116376.&lawCode=HSC
27. https://leginfo.legislature.ca.gov/faces/billTextClient.xhtml?bill_id=201720180SB1263
28. http://www.opc.ca.gov/webmaster/_media_library/2018/06/2018_CA_OceanLitterStrategy.pdf
29. https://nicholasinstitute.duke.edu/plastics-policies/microbeads-toiletries-regulations-sor2017-111
30. https://nicholasinstitute.duke.edu/plastics-policies/restrictions-manufacture-import-and-sale-personal-care-and-cosmetics-products
31. https://nicholasinstitute.duke.edu/plastics-policies/waste-minimisation-microbeads-regulations-2017
32. https://g20mpl.org/partners/republicofkorea
33. https://nicholasinstitute.duke.edu/plastics-policies/environmental-protection-microbeads-england-regulations-2017
34. https://nicholasinstitute.duke.edu/plastics-policies/article-124-law-8-august-2016-reconquest-biodiversity-nature-and-landscapes
35. https://www.ola.org/en/legislative-business/bills/parliament-41/session-1/bill-75
36. https://www.congress.gov/114/plaws/publ114/PLAW-114publ114.pdf
37. https://www.mee.gov.cn/xxgk2018/xxgk/xxgk10/202109/t20210916_945621.html
38. https://g20mpl.org/partners/republicofkorea
39. https://www.ndrc.gov.cn/xxgk/zcfb/tz/202001/t20200119_1219275.html?code=&state=123
40. https://nicholasinstitute.duke.edu/plastics-policies/restrictions-manufacture-import-and-sale-personal-care-and-cosmetics-products
41. https://g20mpl.org/partners/republicofkorea
42. https://www.legifrance.gouv.fr/jorf/id/JORFTEXT000043388114
43. https://www.legifrance.gouv.fr/jorf/id/JORFTEXT000043956924
44. https://g20mpl.org/wp-content/uploads/2022/08/G20MPL-report-2022_2nd-edition_1108.pdf
45. https://nicholasinstitute.duke.edu/plastics-policies/environmental-protection-microbeads-scotland-regulations-2018
46. https://nicholasinstitute.duke.edu/plastics-policies/article-124-law-8-august-2016-reconquest-biodiversity-nature-and-landscapes
47. https://leginfo.legislature.ca.gov/faces/codes_displaySection.xhtml?sectionNum=116376.&lawCode=HSC
48. https://leginfo.legislature.ca.gov/faces/billTextClient.xhtml?bill_id=201720180SB1263
49. http://www.opc.ca.gov/webmaster/_media_library/2018/06/2018_CA_OceanLitterStrategy.pdf
50. https://nicholasinstitute.duke.edu/plastics-policies/microbeads-toiletries-regulations-sor2017-111
51. https://www.ola.org/en/legislative-business/bills/parliament-41/session-1/bill-75
52. https://www.agriculture.gov.au/sites/default/files/documents/national-plastics-plan-2021.pdf
53. https://www.argentina.gob.ar/normativa/nacional/ley-27602-345720
54. https://g20mpl.org/partners/thailand
